# Microfauna–Macrofauna Interaction in the Seafloor: Lessons from the Tubeworm

**DOI:** 10.1371/journal.pbio.0030102

**Published:** 2005-03-15

**Authors:** Antje Boetius

## Abstract

New research and techniques are beginning to provide intriguing clues into the complex relationships that tubeworms form with other species at hydrothermal vents and deep-sea cold seeps

Since their discovery in the 1970s and 1980s, giant tubeworms at hydrothermal vents and cold seeps have fascinated biologists and laymen alike—not only for their alien morphology (Figure 1), but also for epitomizing the perfect animal–microbe symbiosis. They are among the biggest worms on this planet—some over 3 m long—yet they do not eat other organisms. Tubeworms thrive independently of photosynthetic production [1]. They have even lost their entire digestive tract. One of the most exciting findings in early tubeworm research was the discovery that the worm's food is delivered by bacterial symbionts [2]. The chemoautotrophic symbionts live intracellularly in a specialized worm tissue called the trophosome. They are sulfide oxidizers, using the free energy yield from the oxidation of sulfide with oxygen to fix carbon dioxide with their bacterial Rubisco enzyme. In exchange for providing nutrition for the worm, the symbionts are sheltered from grazing, but most importantly, they receive a steady source of sulfide and oxygen via the highly adapted blood circulation system of the worm. (I will never forget how horrified I was as a young student by the amounts of almost human-like blood flowing into my lab dish while dissecting tubeworms to analyze trophosome enzyme activity.) Tubeworm blood physiology, in particular the hemoglobin molecules, are tailored specifically to the needs of the symbionts. However, the host metabolism in itself is not different from that of many other animals, the main source of energy being aerobic respiration of carbohydrates. In other words, tubeworms and their symbionts need oxygen as an electron acceptor—so, after all, they are dependent on photosynthesis, the main oxygen-producing process on earth.

**Figure 1:**
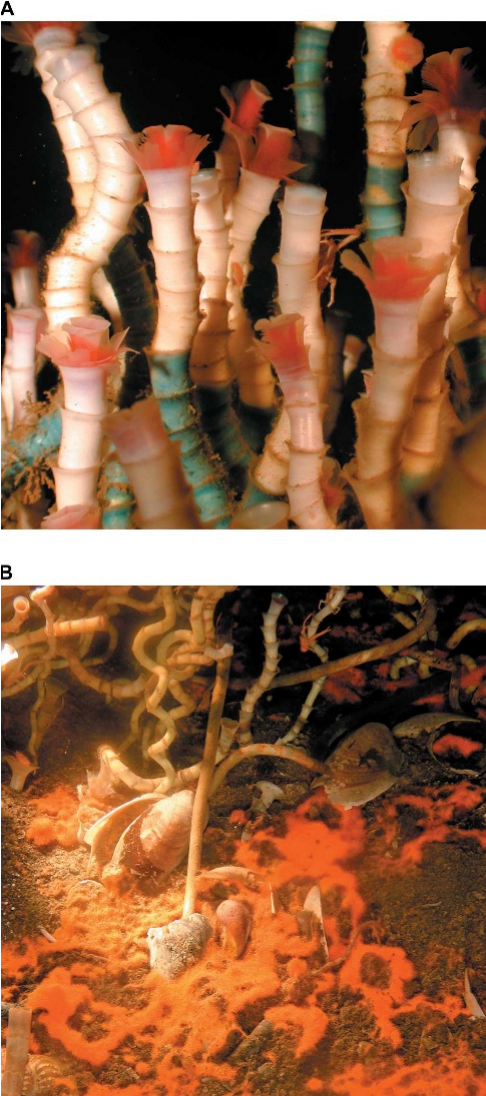
Vestimentiferan Tubeworms (A) Close-up photograph of the symbiotic vestimentiferan tubeworm Lamellibrachia luymesi from a cold seep at 550 m depth in the Gulf of Mexico. The tubes of the worms are stained with a blue chitin stain to determine their growth rates. Approximately 14 mo of growth is shown by the staining here. (Photo: Charles Fisher) (B) Close-up photograph of the base of an aggregation of the symbiotic vestimentiferan tubeworm L. luymesi from a cold seep at 550 m depth in the Gulf of Mexico. Also shown in the sediments around the base are orange bacterial mats of the sulfide-oxidizing bacteria Beggiotoa spp. and empty shells of various clams and snails, which are also common inhabitants of the seeps. (Photo: Ian MacDonald)

## Classification of Host and Symbiont

**Figure 2:**
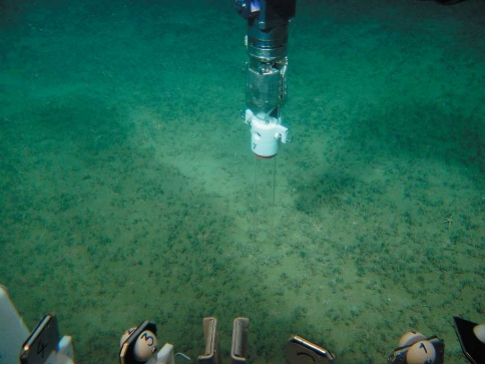
Pogonophoran Tubeworms Being Sampled at the Haakon Mosby Mud Volcano (Source: AWI/IFREMER expedition RV POLARSTERN/VICTOR 6000 in 2003)

With their strange morphology, vent tubeworms were first classified as a novel phylum, Vestimentifera [3]. Recently they have been regrouped together with the pogonophoran tubeworms (Figure 2) into a family of annelid polychaetes called the Siboglinidae [4,5]. Vestimentiferan tubeworms of hydrothermal vents grow on chimneys and other hard substrates in the vicinity of active vents, which emit reduced compounds like hydrogen and sulfide [6]. Vestimentiferan tubeworms living at cold hydrocarbon seeps, i.e., the lamellibrachids and escarpids, are adapted to a sedimentary environment, with a substantial part of the body and tube of many species extending into the mud. All vestimentiferan tubeworms found today at vents, seeps, and a few other reduced submarine habitats harbor sulfide-oxidizing endosymbionts in their trophosome. These symbionts belong to bacteria of the gamma-proteobacteria clade and are phylogenetically related to each other [7]. (For the only known exception see [8].)

## Tubeworm Mysteries

The study of tubeworms is now in its fourth decade, and there are still many fascinating problems to be solved. One of the most interesting—but also most difficult—questions in tubeworm symbiosis is how this obligate and highly integrated interaction between microbes and animals evolved. How can a worm evolve into a perfect home for chemosynthetic bacteria? What are the main evolutionary steps towards this symbiosis, and in which order did they occur? Another intriguing problem is how the worms acquire their endosymbionts, which appear to be taken up from the environment—but so far have not been detected as free-living forms. How does the host recognize its specific symbiont from the vast diversity of gamma-proteobacteria and sulfide oxidizers in the environment? Furthermore, how do tubeworms populate new vents, seeps, and other reducing environments emerging from the ever-changing ocean floor—how do their larvae migrate and settle, and what determines the distribution and lifetime of tubeworm populations in the different mid-ocean ridge and continental margin habitats? Although these questions are still to be answered, new research and techniques are beginning to provide intriguing clues.

## Seep Vestimentifera and Their Energy Source

At some seeps the vestimentiferan tubeworms are so abundant that they form a special habitat that is attractive for a host of other marine species [9]. Seep vestimentiferans are usually thinner, have slower growth rates, and have greater longevity than their vent relatives [10]. For example, a 2-m-long Lamellibrachia luymesi individual is estimated to be more than 200 y old and hence represents the longest-lived animal on earth [11,12]. At seeps, geological processes causing fluid and gas seepage can last hundreds to millions of years, whereas hydrothermal vents often have a lifespan on the order of decades. Vent tubeworm colonies will die when their chimneys stop venting, i.e., delivering sulfide, so they are adapted to a rapidly changing environment, as typified by their fast growth and high reproduction.

Like vent vestimentifera, seep vestmentifera also depend on the availability of sulfide in their direct vicinity, but they are sessile, and anchor on hard substrates such as carbonates. Individual aggregations at seeps can consist of hundreds to thousands of worms, requiring sulfide fluxes of half a mole per day—and this for more than 200 y [12]. So an ecological problem that has always intrigued biologists and geochemists alike is how these tubeworms obtain their energy over the long term. Because vent and seep vestimentifera depend on sulfide-oxidizing symbionts, their distribution is limited to habitats with high sulfide fluxes lasting for at least a few reproductive cycles. However, at cold seeps, unlike hydrothermal vents, most of the chemical energy occurs in the form of hydrocarbons. Cold seeps are characterized by high fluxes of methane, higher hydrocarbons (such as ethane, propane, butane), and/or petroleum from deep subsurface reservoirs. Often the source fluids and gases do not contain much sulfide, because there are no high-temperature seawater–rock interactions involved in their formation, as there are at vents. Some pogonophoran tubeworms at seeps have teamed with methane-oxidizing symbionts to profit from the high availability of hydrocarbons, but seep vestimentiferans do not appear to be able to directly tap this resource. However, seep vestimentiferans are still capable of producing enormous biomass over many years with the help of their sulfide-oxidizing symbionts. So where does the supply of sulfide come from at seeps that enables such large aggregations to be maintained for so long?

Only recently was it realized that anaerobic microbial processes, namely, the oxidation of hydrocarbons with sulfate, could produce astonishingly high fluxes of sulfide in cold seep settings [13,14]. At methane seeps, methanotrophic microbial communities inhabiting the surface sediments oxidize methane with sulfate, which results in very high sulfide fluxes [13]. If the seepage consists of other hydrocarbons such as petroleum, their degradation with sulfate supports an even higher production of sulfide [14]. In some seep sediments, sulfide concentrations can reach 25 mM in subsurface sediments (5–10 cm below the sediment surface). Such concentrations are not known from tubeworm habitats at hydrothermal vents.

However, the zones of high hydrocarbon turnover and sulfide flux at seeps are often limited to only a few centimeters below the seafloor, depending on hydrocarbon flows and the rate of sulfate transport from the bottom water into the sediments. Sulfate is crucial because the free-living hydrocarbon-degrading microbes in seep sediments depend on this electron acceptor for an energy yield. Without sulfate to fuel the oxidation of hydrocarbons, sulfide production stops, even if there is still an enormous reservoir of hydrocarbon available. How might tubeworms, sulfide-oxidizing symbionts, and benthic hydrocarbon degraders overcome these limitations?

## Ménage à Trois—A Model Solution

Cordes et al. [15] have now provided an answer to how the stability of sulfide production is maintained over such long periods and how the worms optimize sulfide uptake. Seep vestimentifera have specific adaptations to their habitat. A main adaptation is the subsurface part of the lamellibrachids called a “root.” The tubeworm root appears to have a special function in the energy cycle of the organism—as in plant roots. Several authors have proposed that the worm roots are not only important in sulfide uptake, but generally in geochemical engineering of the sediments in the direct environment [16,17,18]. Obviously such hypotheses are very difficult to test—today it is still hardly possible to measure gas, petroleum, and sulfide fluxes in the seafloor in situ at depth, especially below tubeworm aggregations. But it is also not possible to recover whole aggregations of worms and to keep them alive in the lab for biochemical and biogeochemical measurements—this would require simulation of seepage under pressure. Instead, Cordes et al. [12,15] have used geochemical and biological modeling to solve the intriguing question of seep vestimentiferan longevity and how they might also interact with free-living anaerobic microbes to increase sulfide availability.

To explain the persistence of the large tubeworm colonies in the Gulf of Mexico, Cordes et al. suggest a broader mutualistic interaction between the tubeworm, its endosymbiont, and benthic hydrocarbon-degrading and sulfide-producing microbes. Seep tubeworms take up sulfide from the sulfide-rich subsurface sediment zones through the roots, but, crucially, they may also release sulfate through the roots as a byproduct of sulfide oxidation by the tubeworm's endosymbiont. Sulfate may also be ventilated through the tube into the sediments. Since anaerobic microbial communities in subsurface hydrocarbon-rich sediments are limited by sulfate influx, any additional supply of sulfate enhances their production of sulfide. Furthermore, the removal of sulfide by the worm will thermodynamically favor anaerobic hydrocarbon oxidation coupled to sulfate reduction. Hence, the tubeworm roots may provide an excellent habitat for anaerobic hydrocarbon oxidizers. For example, Cordes et al. predict in their model that nearly all of the sulfate released through the root will be utilized by benthic microbes for anaerobic hydrocarbon degradation in the direct vicinity of the worm. This process could provide 60% of the sulfide needed by a tubeworm aggregation to persist for 80 y. Hence, it may even be concluded that tubeworms farm anaerobic hydrocarbon degraders to provide a steady supply of sulfide to their endosymbionts. Especially at petroleum seeps, this would guarantee a lifelong energy source and help explain the extraordinary longevity of the worms. The mutual benefit arising from the association of sulfide oxidizers, sulfate reducers, and a host worm is known to be exploited by the oligochaete Olavius algarvensis [19]. In this very effective “ménage à trois” the sulfate reducer has even become an endosymbiont of the worm. Interestingly, some of our recent studies at the methane seeps of Hydrate Ridge (Cascadia margin) also show that certain populations of anaerobic methane oxidizers are specifically associated with seep organisms—such as the symbiotic clam Calyptogena and the giant filamentous sulfide oxidizer Beggiatoa [20]. But many more examples may be out there, of bacterial and archaeal populations specifically growing in the “rhizosphere” of benthic organisms, potentially profiting from bioturbation, bioirrigation, fecal deposits, and exudates.

**Figure 3:**
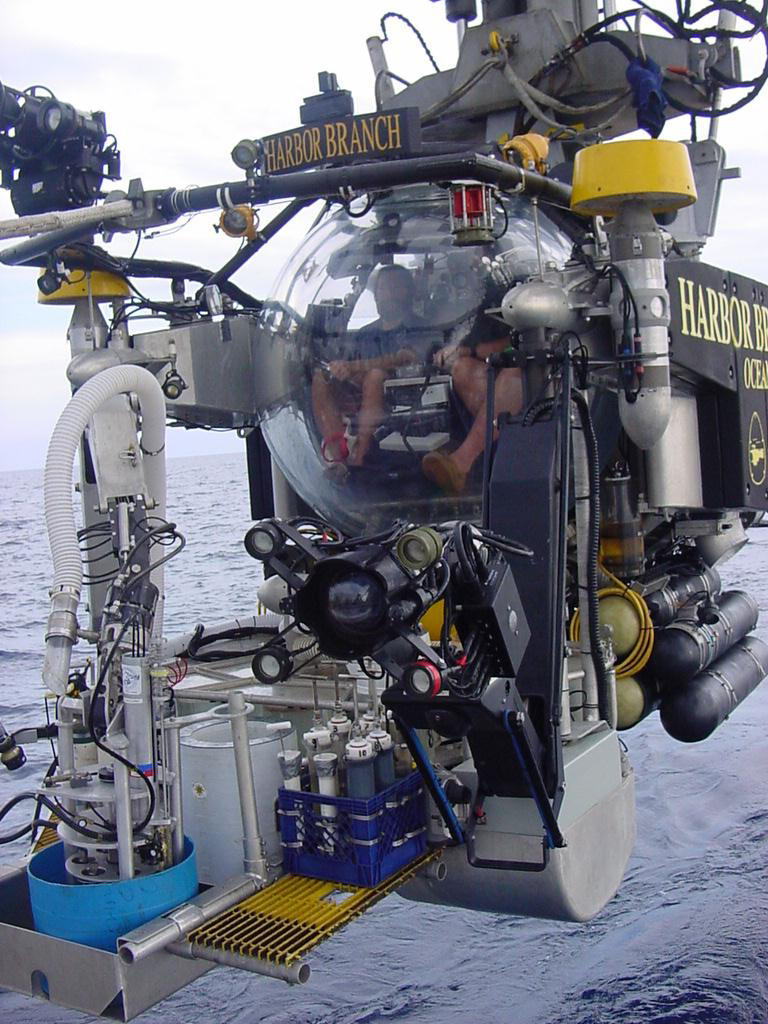
Harbor Branch Oceanographic Institution's Submersible “Johnson SeaLink” (Source: Gulf of Mexico Cruise SJ0107)

The association and interaction between benthic fauna and sedimentary microorganisms is a very interesting field of study, although inevitably still very speculative. So far it has been limited by a lack of appropriate technologies, not only for in situ biogeochemical and biological measurements, but also for quantitative investigation of specific functional microbial populations. Some insight can be provided by clever environmental modeling approaches—such as the one developed by Cordes et al., but ultimately the models need empirical verification. Only very recently has it become possible to combine visually targeted sampling (Figure 2) and high-resolution measurements of geochemical gradients with molecular tools for the identification of microbes, such as 16S rDNA and organic-biomarker-based techniques. For the study of continental margin and deep-sea ecosystems, this requires the availability of underwater vehicles (Figure 3) as well as multidisciplinary research platforms and extensive, highly detailed lab work—so this is very expensive research. Yet this is the future, if we want to determine whether such an intriguing ménage à trois as proposed by Cordes et al. accounts for the presence and longevity of these extraordinary tubeworms, and possibly also other chemosynthetic symbioses, forming some of the most fascinating marine ecosystems at continental margins.
